# Additional Intra- or Inter-session Balance Tasks Do Not Interfere With the Learning of a Novel Balance Task

**DOI:** 10.3389/fphys.2018.01319

**Published:** 2018-09-19

**Authors:** Louis-Solal Giboin, Markus Gruber, Andreas Kramer

**Affiliations:** Sensorimotor Performance Lab, University of Konstanz, Konstanz, Germany

**Keywords:** contextual interference, rehabilitation, motor learning, varied practice, sensorimotor training, specificity, retrograde interference

## Abstract

**Background:** It has been shown that balance training induces task-specific performance improvements with very limited transfer to untrained tasks. Thus, regarding fall prevention, one strategy is to practice as many tasks as possible to be prepared for a multitude of situations with increased fall risk. However, it is not clear whether the learning of several different balance tasks interfere with each other. A positive influence could be possible via the contextual interference (CI) effect, a negative influence could be induced by the disruption of motor memory during consolidation or retrieval.

**Methods:** In two 3-week training experiments, we tested: (1) whether adding an additional balance task in the same training session would influence the learning of a balance task [first task: one-leg stance on a tilt-board (TB), six sessions, 15 × 20 s per session; additional task: one-leg stance on a slack line (SL), same amount of additional training]; (2) whether performing a different balance task (SL) in between training sessions of the first task (TB) would influence the learning of the first task. Twenty-six healthy subjects participated in the first experiment, 40 in the second experiment. In both experiments the participants were divided into three groups, TB only, TB and SL, and control. Before and after the training period, performance during the TB task (3 × 20 s) was recorded with a Vicon motion capturing system to assess the time in equilibrium.

**Results:** Analyses of variance revealed that neither the additional intra-session balance task in experiment 1 nor the inter-session task in experiment 2 had a significant effect on balance performance improvement in the first task (no significant group × time interaction effect for the training groups, *p* = 0.83 and *p* = 0.82, respectively, only main effects of time).

**Conclusion:** We could not find that additional intra- or intersession balance tasks interfere with the learning of a balance task, neither impairing it nor having a significant positive effect. This can also be interpreted as further evidence for the specificity of balance training effects, as different balance tasks do not seem to elicit interacting adaptations.

## Introduction

Falls have a major impact on the quality of life, especially in aged people and patients suffering from motor neuronal disorders (Sattin et al., 1990; Pavol et al., 2002; Gunn et al., 2013; Kim et al., 2013). Indeed, a fall for these populations is often a factor of hospitalization, immobilization, loss of autonomy, and increased mortality (Hannan et al., 2001; Jiang et al., 2005; Alvarez-Nebreda et al., 2008). One strategy to reduce fall occurrence consists in improving the balance of at risk populations (Gillespie et al., 2012). The success of this strategy is increased when the training is designed specifically to compensate pathological deficits or physical weaknesses occurring through the aging process (Horak et al., 2009; Gillespie et al., 2012). In addition, training one balance task seems to have quite task-specific effects (Giboin et al., 2015; Kummel et al., 2016; Donath et al., 2017). As one could encounter many types of balance perturbations in daily activities that could potentially lead to falls, a large spectrum of balance tasks should be included in the training. However, because of the reduced fitness abilities, but also the lack of motivation or time constraints of at risk populations, the number of tasks that can be trained, the overall volume and the time devoted to such training is very limited (Child et al., 2012; Khan et al., 2014; Mohler et al., 2014). Hence a very strong need to optimize the training sessions. Despite an extensive amount of scientific literature on motor learning and on the effects of balance training, less importance has been given to the ways of optimizing the motor learning of balance tasks. In particular, it is not clear whether the learning of two different balance tasks can affect the outcome, positively or negatively, of a short-term balance training.

Indeed, there is some evidence showing that variable practice, i.e., learning several tasks with a random or serial schedule during the training session, induces a lower acquisition but a better retention than constant training, i.e., learning only one task (Shea and Morgan, 1979; Shea and Kohl, 1990; Shea et al., 1990; Sekiya et al., 1996). This effect, called contextual interference (CI), is probably generated by the more difficult learning situation of the varied practice (Magill and Hall, 1990). Since CI effect may not be generalizable to every type of tasks (Magill and Hall, 1990; Brady, 2008), it seems of a great interest to test whether variable practice can enhance the learning of a balance task.

On the other hand, there is evidence from several fundamental motor learning studies that learning can be hindered and negative interference can occur when subsequently learning two or more novel motor tasks (Brashers-Krug et al., 1996). It has been proposed that when practicing a novel motor task, the motor memory of this task is unstable and can be disrupted by practicing another motor task (Shadmehr and Brashers-Krug, 1997), especially if the second task is quite similar (Lundbye-Jensen et al., 2011). The results of several visuomotor rotation and force-field adaptation studies suggest that this is not only the case when the second task is practiced shortly after the first one, but also when it is performed a day or even a week later (Caithness et al., 2004).

Thus, in the present study, practicing one balance task was compared to practicing the same balance task and an additional balance task, either in the same session or in a different session. In an applied setting, we wanted to assess whether adding the second balance task would facilitate learning as predicted by the CI framework, or on the contrary whether this would hinder learning as some fundamental motor learning and consolidation studies suggest.

## Materials and Methods

### Participants

The experiments were approved by the local ethics committee and were in accordance with the latest revision of the Declaration of Helsinki. All participants gave written informed consent before starting the experiment. The 69 subjects were recruited among sport students and divided so 26 (15 males, 11 females, age 26 ± 7 years, height 176 ± 11 cm, body mass 74 ± 15 kg) of them participated in the experiment 1 and 40 (three drop-outs unrelated to the study, 32 male, 8 female, age 23 ± 3 years, height 180 ± 9 cm, body mass 74 ± 12 kg) of them participated in the experiment 2. All subjects had to be naïve to the test and training tasks.

### Experiment 1: Additional Intra-session Task

**FIGURE 1:**
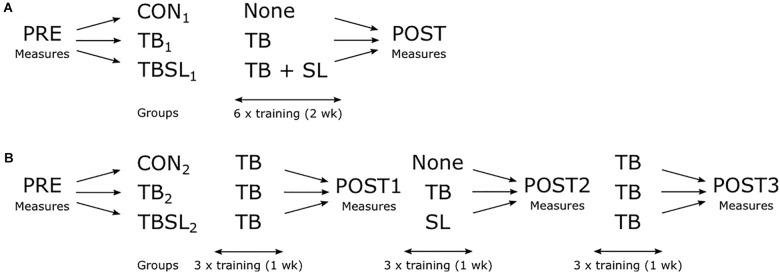
Experimental design. **(A)** Experiment 1. After pre-practice measurements, subjects were divided into three groups (CON_1_: no training, TB_1_: tilt board training, TBSL_1_: tilt board and slack line training). After six training sessions, post-practice measurements were done. **(B)** Experiment 2. After pre-practice measurements, subjects were divided into three groups. During week 1, all three groups trained with the tilt board. During week 2, CON_2_ did not train, TB_2_ trained with the tilt board, and TBSL_2_ trained with the slack line. During week 3, all three groups trained with the tilt board. Measurements were done after week 1, 2, and 3.

The 26 subjects were divided in three groups: tilt board group (TB_1_, *N* = 9), tilt board and slack line group (TBSL_1_, *N* = 9), and control group (CON_1_, *N* = 8). All subjects did a PRE and a POST practice measurement on the tilt board. The subjects were divided into the three groups after the PRE measurements so their initial balance ability on the tilt board was matched. The subjects from the TB_1_ group practiced with only the tilt board for six sessions (three sessions per week for 2 weeks, at least one day of rest in between sessions), the TBSL_1_ subjects had the same amount of tilt board practice as the TB_1_ group, but in addition slack line practice during the same practice session for six sessions, and the subjects from CON_1_ did not train, see also **Figure 1A**. The practice for TB_1_ during each session consisted of 15 trials of 20 s on the tilt board separated by 10 s of rest. The practice of TBSL_1_ consisted of 30 trials of 20 s, alternating between one trial on the tilt board and one trial on the slack line, also with 10 s of rest in between. Every five trials were separated by 1 min of rest.

### Experiment 2: Inter-session Task

The 40 subjects that participated in the second experiment were divided into three groups: tilt board group (TB_2_, *N* = 12), tilt board and slack line group (TBSL_2_, *N* = 14), and control group (CON_2_, *N* = 14). All subjects did a PRE and several POST measurements on the tilt board (after the third [POST1], the sixth [POST2] and the final practice session [POST3]). The subjects were divided into the three groups after the POST1 measurements so their initial balance performance on the tilt board during PRE and POST1 was matched. For all groups, the practice period lasted 3 weeks with 3 practice sessions per week (total of 9 practice sessions for TB_2_ and TBSL_2_, 6 sessions for CON_2_). The subjects in TB_2_ practiced only with the tilt board for the whole 3 weeks. The subjects in TBSL_2_ practiced with the TB during week 1 and week 3, but during week 2 they practiced with the slack line instead of the tilt board. The subjects in CON_2_ also practiced with the tilt board during week 1 and 3, but did not train at all during week 2, see also **Figure 1B**. The practice with the tilt board consisted of 20 trials of 20 s separated by 10 s of rest. The practice with the slack line consisted of 20 trials of 20 s separated by 10 s of rest. Every five trials were separated by 1 min of rest.

### Balance Tasks

Whatever the balance task, each subject had to balance on the preferred leg (always the same during the whole experiment), with hands on the hip.

**FIGURE 2:**
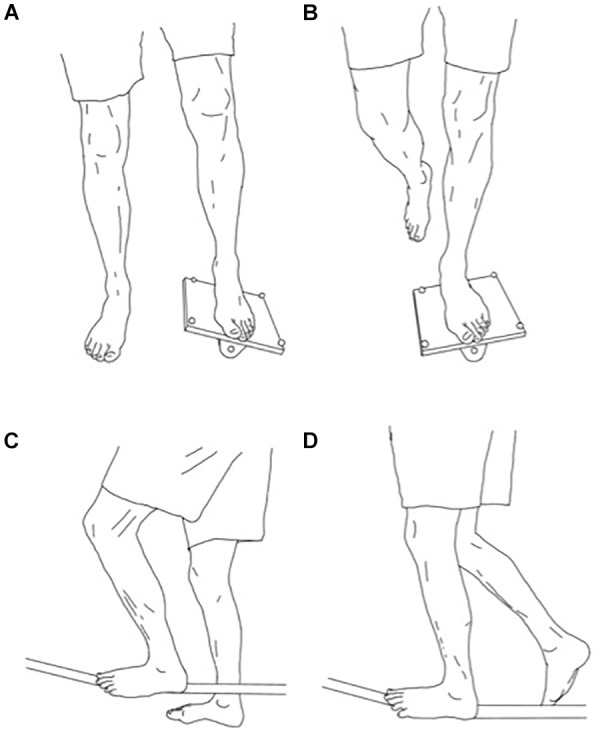
Illustrations of the TB and SL tasks. **(A)** Starting position for the TB task. **(B)** Equilibrium position for the TB task. **(C)** Starting position for the SL task. **(D)** Equilibrium position for the SL task.

The tilt board was custom-made and consisted of a wooden platform (25 cm × 25 cm × 1 cm) mounted on a semi-circular wooden structure with a height of 6.5 cm. The aim was to bring and maintain the platform of the tilt board into a horizontal position while standing on it with one leg. The tilt board was oriented so the axis of the semi-circular wooden structure was in parallel with the longitudinal axis of the foot. At the beginning of each trial (measurement or practice), the tilt board was always positioned with the same edge on the floor, with the preferred leg on the tilt board and the other leg firmly on the ground. Then, the subject had to lift this leg off the floor (**Figure 2A**) prior to bringing the platform of the tilt board into a horizontal position (**Figure 2B**). When the subject had to touch the ground with the free leg, i.e., to prevent a fall, this whole procedure was started again.

The slack line (Gibbon slacklines, Classic Line X13, width of 5 cm) was secured between two pillars separated by 5.6 m, at a height of 60 cm, with a mark in the middle of the line where the participants had to get on the slack line and try to balance them. Participants started each trial by standing on the side of the line, with the preferred leg on the line and the other leg standing firmly on the ground (**Figure 2C**). Then, the subject had to lift his leg off the ground and try to balance himself with only one foot on the slack line, while limiting the lateral oscillations of the slack line (**Figure 2D**).

During the practice and the measurements, investigators controlled and if necessary corrected the execution of the balance tasks.

### PRE and POST Measurements

The PRE and POST measurements were done on the tilt board. They consisted of 5 trials of 20 s separated by 10 s of rest. The first 2 trials served as familiarization trials and only the last 3 were used to quantify the performance of each subject. POST measurements were done after at least one day of rest after the last practice session.

### Data Collection and Analysis

Four reflective markers were placed on the corners of the tilt board, so that the position and angle of the board could be recorded and analyzed with a motion capture system at 200 Hz (Vicon Nexus, 12 T-series T40s cameras). The performance during a trial was evaluated by calculating the amount of time the tilt board was within a margin of ±5° of the horizontal position [i.e., when the angle of the board with respect to the floor was between -5° and + 5°, see (Giboin et al., 2015)]. The performance was defined as the mean of the three recorded trials.

### Statistics

Statistical tests were done with JASP (Version 0.8.3.1, University of Amsterdam). For both experiments, the changes in performance after the practice were assessed with a mixed design analysis of variance (ANOVA) with repeated measures, using time as repeated measure and group as between-subject factor.

## Results

### Experiment 1: Additional Intra-session Task

**FIGURE 3:**
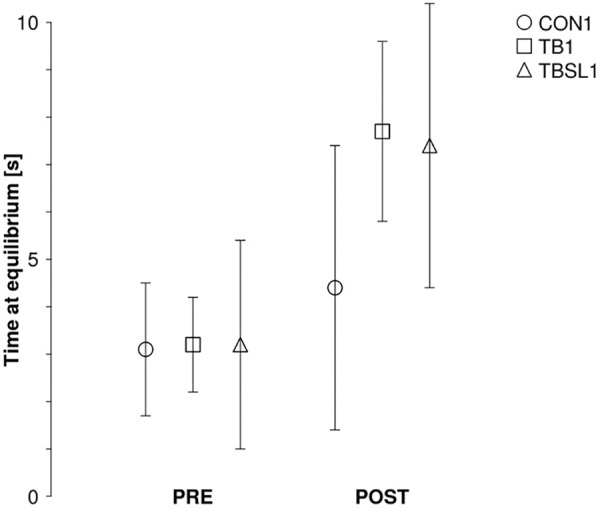
Experiment 1, intra-session varied practice. Grouped data of the average performance in seconds on the TB from CON1 (circle, no training), TB1 (square, training with TB), and TBSL1 (triangle, training with TB, and SL) before (PRE) and after (POST) the six training sessions. Error bars represent standard deviations.

As depicted in **Figure 3**, performance during the tilt board task increased after the six training sessions for TB_1_ and TBSL_1_. Indeed, when considering only the two training groups, the ANOVA showed no significant group^∗^time effect (*F*_1,16_ = 0.05, *p* = 0.83), only a significant main effect of time (*F*_1,16_ = 65.6, *p* < 0.001). When adding the control group to the analyses to ensure that the time effect was not only due to test–retest improvements, there was a significant group^∗^time effect (*F*_2,23_ = 5.66, *p* = 0.01).

### Experiment 2: Inter-session Task

**FIGURE 4:**
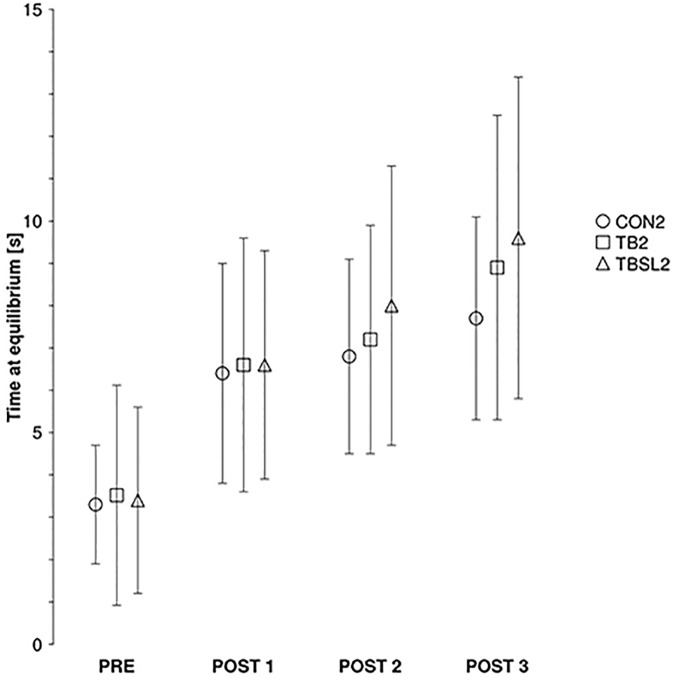
Experiment 2, inter-session varied practice. Grouped data of the average performance in seconds on the TB from CON2 (circle, no training during week 2), TB2 (square, training with TB during the 3 weeks) and TBSL2 (triangle, training with SL during week 2) before (PRE), after 3 training sessions (POST1), 6 training sessions (POST2), and 9 training sessions (POST3). Error bars represent standard deviations.

As depicted in **Figure 4**, the performance in the three groups increased similarly after the 3 weeks of training. When considering only the two training groups, the ANOVA showed no significant group^∗^time effect (*F*_3,72_ = 0.31, *p* = 0.82), only a significant main effect of time (*F*_3,72_ = 33.7, *p* < 0.001). When adding the control group to the analyses, there was no significant group^∗^time effect (*F*_6,111_ = 0.62; *p* = 0.7), only a significant main effect of time (*F*_3,111_ = 49.76; *p* < 0.001).

## Discussion

In this study, we found no evidence for a positive or negative interference on the learning of a novel balance task when adding a second balance tasks, neither when doing so in the same session nor in between sessions.

### Additional Intra-session Balance Task

The addition of an intra-session balance task, i.e., variable practice, did not improve performance in the balance task to a greater extent than practicing just one novel balance task, i.e., constant practice, despite the double amount of total training volume. This lacking difference cannot be explained by a ceiling effect, since the measured performance remained lower than performance measured in experiment 2 after 3 weeks of training. However, the absence of effect from CI and variable practice is not unheard of when tested with complex whole-body tasks instead of for instance with simple pointing tasks (for review, see Brady, 2008). Several hypotheses may explain the absence of CI effect. First, because of the complexity of both tasks, the CI effect may have been too high and possibly induced an “overload” of the processing requirements, reducing the learning efficiency, especially at the beginning of the training but no more at a later stage, hence no difference with the constant practice group (Shea et al., 1990). Second, the balance task with the tilt-board (TB) may itself induce a high level of CI due to its complexity (Albaret and Thon, 1998). Indeed, as the board responds already to small bodyweight shifts, a large panel of different balance perturbation and balance recovery movement sequences can be seen throughout one training session. This variety of postural movements may by itself elicit a strong CI effect, which may already saturate the possible retention increase effect induced by high CI even with the addition of another task (Albaret and Thon, 1998). Finally, it must be noted that even if no effect of retention was seen on the learning of a balance task after a short-term training, there was also no impairment observed. Indeed, it has been reported that intra-session practice of similar tasks could, when performed with a block design, induce retrograde interference. The resulting impairment in learning is possibly caused by memory consolidation disruption. The practice of two very similar tasks would in theory prevent synaptic change stabilization since both tasks share the same underlying neural networks (Lundbye-Jensen et al., 2011). In the present study, the lack of increase or impairment in the learning of the balance task despite the varied practice supports the concept that balance is more a sum of specific skills than a general ability (Giboin et al., 2015; Ringhof and Stein, 2018). Indeed, the lack of altered learning implies that both tasks were possibly too different to induce a CI effect, or alternatively, too different to share the same underlying neural networks and impair memory consolidation. In conclusion, even though no gain in learning efficiency could be found, the variable practice can still be recommended for short-term balance training if the goal is to learn several balance tasks.

### Additional Inter-session Balance Task

The second experiment served a twofold purpose: investigate possible negative retrograde interference effects in a very applied setting, possibly caused by motor memory consolidation disruption (see above), and assess potential differences due to the timing of practice of the additional task: motor learning studies have shown that directly after learning, the motor memory of the task that was just practiced is still fragile, i.e., not consolidated yet (Brashers-Krug et al., 1996). If this phenomenon played an important role in the acquisition of a novel balance task, it should have been reflected in the results of the first experiment, where after each trial of the first task, a trial of the second–possibly interfering–task was performed. This was not the case, but retrograde interference has also been reported after days and even a week in visuomotor rotation and force field adaptation tasks (Caithness et al., 2004). To test the practical influence of this re-engagement of the motor memory that potentially makes it vulnerable to disruption again, we performed the second experiment. The results indicate that this effect does not seem to play an important role in the acquisition of novel balance tasks. If anything the group that practiced an additional novel balance task (TBSL_2_) performed even slightly better in the first task, even though they did not train the first task during that time, in contrast to the other group (TB_2_) that continued to practice the first task. This slight advantage of adding a second balance task might be explained by motivational issues, as practicing the same balance task for 3 weeks might be considered less exciting by some participants than when it is interspersed with 1 week of practicing a different novel task. In any case, the retrograde interference effect described for some motor learning studies using visuomotor rotation or force-field adaptation tasks was not observed in our study, indicating that this effect does either play a minor role in such an applied setting with complex whole-body tasks, and that other factors such as motivation play a more important role.

It is interesting to note that in the control group that did not train during the second week, the performance did not differ compared with the two training groups. However, robust recalls of a discrete motor task, even after longer retention test interval, have already been documented (Dail and Christina, 2004; Savion-Lemieux and Penhune, 2005). Moreover, we suggest that the slow learning phase was already reached after three training sessions. Then, at this stage of learning, one more week of training may not be sufficient enough to induce significant improvements, which could explain the lack of performance difference between the three groups. Thus, it must be noted that a possible performance difference could possibly appear with longer duration training.

## Conclusion

The results of this study suggest that adding an additional distinct balance task within or in between practice sessions does not interfere with the motor learning of a novel balance task. The lack of interference, positive or negative, support the concept that balance training elicits mostly distinct task-specific adaptations that do not interfere with each other. From an applied perspective, sequentially practicing several balance tasks for rehabilitation or fall prevention can be recommended, as the additional tasks do not seem to hinder learning.

## Author Contributions

AK, MG, and L-SG conceptualized the study. AK and L-SG collected, analyzed, and interpreted the data. L-SG drafted the manuscript. AK and MG revised the draft.

## Conflict of Interest Statement

The authors declare that the research was conducted in the absence of any commercial or financial relationships that could be construed as a potential conflict of interest.
